# Autochthonous Human Fascioliasis, Belgium

**DOI:** 10.3201/eid2601.190190

**Published:** 2020-01

**Authors:** Sandrine Milas, Camelia Rossi, Ivan Philippart, Pierre Dorny, Emmanuel Bottieau

**Affiliations:** University Hospital Center Tivoli, La Louvière, Belgium (S. Milas);; University Hospital Center Ambroise Paré, Mons, Belgium (C. Rossi);; Regional Hospital Mons-Hainaut, Mons (I. Philippart);; Institute of Tropical Medicine, Antwerp, Belgium (P. Dorny, E. Bottieau)

**Keywords:** Human fascioliasis, triclabendazole, lymnaeid snails, cattle, Fasciola, zoonoses, parasites, Belgium, Fasciola hepatica

## Abstract

We report 2 cases of human fascioliasis (HF) in Belgium, likely caused by consumption of vegetables from a garden that was flooded by pasture runoff. Because autochthonous HF is rare and the route of transmission was unusual, HF was not diagnosed until 6 months after symptom onset in both cases.

Human fascioliasis (HF) is a plantborne and waterborne infection caused by the trematodes *Fasciola hepatica* in temperate areas and *F. gigantica* in tropical areas (*1*,*2*). *Fasciola* spp. trematodes infect herbivorous mammals and humans. The *Fasciola* life cycle requires 2 hosts; ruminants carry adult worms and excrete eggs into the environment in feces; lymnaeid snails are invaded and release cercariae, which encyst as metacercariae on aquatic vegetation. Humans become infected by ingesting raw aquatic vegetables or consuming plants or water containing metacercariae (*3*). Symptoms of fascioliasis are stage-specific and related to hepatic migration by larva or obstruction of the biliary ducts by adult worms (*1*,*2*).

In Belgium, only 6 cases of HF have been published since 1960 (*4*–*6*). We describe 2 autochthonous cases of HF. The cases were seen in different hospitals and initially were not linked epidemiologically. 

Case 1 was in a 72-year-old man with no underlying medical conditions and no history of travel outside the country who was referred to the Regional Hospital of Mons in November 2008. He had fever, abdominal pain, rash, and hypereosinophilia that had lasted for 8 weeks (Table). Fecal egg detection was negative. Several serologic tests targeting parasitic infections were performed (Table); results for *Trichinella spiralis* were positive, but this diagnosis was discarded in the absence of myalgia and elevated creatine kinase. Eventually, a diagnosis of idiopathic hypereosinophilic syndrome was made. The patient received high doses of corticosteroids, but his condition did not improve. He was reevaluated in March 2009, and HF was considered on the basis of combined clinical, laboratory, and radiologic findings (Table). An indirect hemagglutination test for *Fasciola* spp. was performed by using ELI.HA Distoma (ELITech Group, https://www.elitechgroup.com), and results were positive. The patient received triclabendazole (10 mg/kg/d) for 2 consecutive days. His symptoms abated, and his eosinophil count was nearly normal 1 month later (Table).

**Table:** Clinical, laboratory, and radiologic features of 2 autochthonous cases of human fascioliasis, Mons, Belgium, 2008–2009*

Features	Case 1	Case 2
Patient age, y/sex	72/M	59/F
Date of initial examination	Nov 2008	Dec 2008
Laboratory tests, date		
Eosinophil cell count/mL (% leukocytes)†		
Nov 2008	9,688 (56)	ND
Dec 2008	7,504 (56)	6,750 (43)
Mar 2009	8,830 (56.6)	8,525 (55)
1 mo after triclabendazole	627 (10.8)	3,526 (41)
5 mo after triclabendazole	512 (8.4)	812 (14.5)
Aspartate aminotransferases, IU/L‡		
Nov 2008	53	ND
Dec 2008	30	21
Mar 2009	25	23
1 mo after triclabendazole	26	32
5 mo after triclabendazole	17	ND
Serologic test (method), date	Dec 2008	Jan 2009
*Ascaris* spp. (immunodiffusion)	–	–
*Echinococcus granulosus* (ELISA)	–	–
*Echinococcus multilocularis* (ELISA)	–	ND
*Toxocara* spp. (ELISA)	–	–
* Taenia solium*	ND	–
*Trichinella spiralis* (ELISA)	+	–
*Fasciola* (indirect hemagglutination), Mar 2009§	1/640	1/2,560
1 mo. after triclabendazole	1/640	ND
5 mo. after triclabendazole	1/320	1/320
Abdominal CT, date	Nov 2008, multiple hypodense liver nodules	Mar 2009, multiple hypodense liver nodules
Liver biopsy, date	Dec 2008, chronic active hepatitis with acute necrosis, presence of eosinophils in portal spaces	ND
*Both case-patients had an early acute phase of disease during Sep–Dec 2008 with high fever, abdominal pain, generalized itching, and urticarial skin rash. Both cases then had a later disease phase during Nov 2008–Mar 2009 with persistent fatigue and weight loss. CT, computed tomography; IU, International Units; ND, not done; –, negative; +, positive. †Normal values, 0–400/mL (<7%). ‡Normal values 10–40 IU/L. §Normal value <1/320.		

Case 2 was in the index case’s neighbor, who experienced similar symptoms that lasted for 3 months before she was seen at the University Hospital Center Ambroise Paré in Mons in December 2008. She also was misdiagnosed initially (Table). In February 2009, 2 stool examinations were negative for parasite eggs. In March, an indirect hemagglutination test for *Fasciola* was performed and was highly positive. The patient received a single dose of triclabendazole (15 mg/kg) and recovered fully within 5 months (Table).

A detailed anamnesis revealed that both patients consumed unwashed raw vegetables from case-patient 2’s garden, which was flooded with runoff from a neighboring cattle pasture in August 2008. We hypothesize the vegetables were contaminated by metacercariae, either by *Fasciola*-infested amphibious snails washed into the garden or directly by runoff. We did not perform sampling of the garden. Cases related to garden vegetables contaminated by flooding have been reported previously, such as in Corsica (*3*).

HF is not a notifiable disease in Belgium. Among 6 published cases, 3 occurred in a cluster related to consumption of homegrown watercress, and 3 nonclustered cases had a questionable autochthonous nature (*4*–*6*). Consumption of watercress and dandelions is uncommon in Belgium, but common in France, where ≈300 HF cases occur annually (*7*). However, *Fasciola* infection in cattle is common in Belgium; herd prevalence is 37.3% in Flemish dairy cattle (*8*). In addition, a 2008 survey of snails showed 1.31% of *Galba truncatula* and 0.16% of *Radix* spp. were infected by *F. hepatica* trematodes (*9*).

Both cases in this study experienced an acute invasive stage and a considerable delay in HF diagnosis. Clinicians should be aware of key elements of HF, including potential diet exposure, clinical signs and symptoms, and imaging and laboratory findings. Contrast-enhanced computed tomography scans of the liver sometimes show tortuous subscapular tracts associated with hypodense nodules and hepatomegaly during the acute phase (*2*). In industrialized countries, human cases occur singly or in small clusters, and diagnosis usually is made during the invasive phase by combined clinical, laboratory, and imaging findings. Serologic tests can detect antibodies within 2 weeks after infection but might have low specificity. *Fasciola* eggs can appear in stool 3–4 months postinfection, depending on the infection burden and the ability of the flukes to produce eggs. Intermittent shedding can occur (*1*,*2*). Co-proantigens are detectable 8 weeks after infection and have a high sensitivity, but 1 negative result despite high egg shedding has been reported (*2*).

Triclabendazole, licensed in Europe only by Novartis (https://www.novartis.com), at 10–15 mg/kg/day in 1 dose or on 2 consecutive days, is the preferred treatment, and patients usually recover rapidly. Resistance increasingly is described in ruminants and treatment failures have been reported in humans (*2*,*10*).

Although overlooked in countries in northern Europe, HF should be considered in cases of unexplained eosinophilia associated with liver symptoms, even in the absence of ingestion of freshwater plants. This zoonotic condition highlights the need for good epidemiologic communication between human and animal health workers.
